# CD40L membrane retention enhances the immunostimulatory effects of CD40 ligation

**DOI:** 10.1038/s41598-019-57293-y

**Published:** 2020-01-15

**Authors:** Taha Elmetwali, Asmaa Salman, Wenbin Wei, Syed A. Hussain, Lawrence S. Young, Daniel H. Palmer

**Affiliations:** 1 Institute of Translational Medicine, Department of Molecular and Clinical Cancer Medicine, University of Liverpool, 2nd Floor Sherrington Building, Ashton Street, Liverpool, L69 3GE UK; 2 Present Address: National Research Centre, 12662 Dokki, Giza Egypt; 3 Institute of Immunology and Immunotherapy, University of Birmingham, Birmingham, B15 2TT UK; 4 Department of Biosciences, Durham University, Durham, DH1 3LE UK; 5 Sheffield Academic Unit of Oncology Department of Oncology and Metabolism Medical School, University of Sheffield, Sheffield, S10 2 RX UK; 6 Warwick Medical School, University of Warwick, Coventry, CV4 7AL UK; 7 Clatterbridge Cancer Centre NHS Foundation Trust, Clatterbridge Road, Bebington, Wirral, CH63 4JY UK

**Keywords:** Translational immunology, Targeted therapies

## Abstract

In carcinomas, the nature of CD40 ligand shapes the outcome of CD40 ligation. To date, the consequences of membrane-bound CD40L (mCD40L) on its immune-stimulatory function are unknown. Here, we examined the impact of mCD40L versus soluble CD40L (sCD40L) on T24 bladder carcinoma gene expression profiling. Of 410 differentially expressed genes, 286 were upregulated and 124 downregulated by mCD40L versus sCD40L. Gene ontology enrichment analysis revealed immune-stimulatory function as the most significant enriched biological process affected by upregulated transcripts, while those downregulated were critical for cell growth and division. Furthermore, immature dendritic cells (iDC) responded to mCD40L with enhanced maturation and activation over sCD40L evidenced by higher expression levels of CD83, CD86, HLA-DR and CD54, increased secretion of IL12 and IL10 and higher tumour-antigen (TA) uptake capacity. Furthermore, autologus CD3+ T cells responded to TA-loaded mCD40L-activated DC with increased proliferation and cytotoxic response (CD107a and IFN-γ-producing CD3+ CD8+ T cells) to the tumour-loaded autologous PBMCs compared to sCD40L. Thus, these data indicate that mCD40L enhances the immunostimulatory capacity over sCD40L. Furthermore, the ability of mCD40L to also directly induce cell death in CD40-expressing carcinomas, subsequently releasing tumour-specific antigens into the tumour microenvironment highlights the potential for mCD40L as a multi-faceted anti-cancer immunotherapeutic.

## Introduction

The fundamental role of the CD40 receptor, a member of the TNFR superfamily together with its ligand (CD40L/CD154) in co-ordinating immune responses has been widely recognised as an early event in initiating immune responses^1^. CD40, a 40-kD type 1 transmembrane protein is expressed in normal B cells, malignant hematopoietic cells and antigen presenting cells (APC), including dendritic cells (DC) and monocytes^2,3^. Furthermore, activated CD4 + and CD8+ T cells also express CD40 receptor, where in absence of CD40 expression, CD8+ T cells were unable to differentiate into memory cells or receive CD4 help^4^. In addition to hematopoietic cells, several carcinomas express CD40 receptor, including those of the ovary, liver, and bladder, despite receptor expression being undetectable in normal epithelium derived from the same tissue^5^. In contrast to the wide-spectrum of CD40 expression, the expression of CD40L, a 32-kD protein is restricted mainly to activated CD4+ T cells and, to a lesser extent, activated B cells and platelets^6^. In hematopoietic cells the importance of CD40-CD40L interaction is well-recognized particularly in shaping adaptive immune responses, where the outcome of CD40 ligation is cell-type dependant, with CD40 activation in DC leading to differentiation into IL12- and IFNγ-secreting cells and upregulation of co-stimulatory and adhesion molecules; while B cells respond by Ig-class switching^7^. Furthermore, the strength of CD40 ligation by CD40L can influence the signalling outcome, with strong CD40 ligation enhancing antigen processing and presentation in Burkitt’s lymphoma cells, compared to weak CD40 ligation^8^.

Like other TNF family ligands, CD40L is naturally expressed as membrane-bound molecules that undergo cleavage from the membrane into a soluble form upon binding with the CD40 receptor via disintegrin and metalloproteinases^9,10^. The membrane-bound and the soluble forms of CD40L also differentially influence the outcome of CD40-CD40L interaction in carcinomas, with membrane-bound CD40L (mCD40L) directly inducing apoptosis^11–13^, whilst a blockade of the protein synthesis machinery is a prerequisite for cell death induction by soluble CD40L (sCD40L)^14^. Thus the outcome of CD40-CD40L interaction is not only determined by the cell type but also by the strength and the mode of CD40 ligation.

We have previously generated a mutant form of CD40L that is resistant to proteolytic cleavage, with retained expression at the cell membrane and we have shown that this mCD40L can induce apoptosis in CD40-expressing carcinomas, indicating its potential as an anti-cancer therapy. Given the widespread expression of CD40 in immune cells, it is important to characterise its effects on immune cells and immune regulation to better understand the potential of mCD40L as a cancer therapeutic. The current study addresses these issues and confirms the potential of mCD40L as a multi-faceted anti-cancer therapeutic with the capability to directly induce cancer cell apoptosis, at the same time robustly inducing several elements of the immune response, suggesting potential advantages over other immunostimulatory approaches.

In this study, we first sought to investigate the gene expression profile following CD40 activation by either mCD40L delivered by replication-deficient recombinant adenoviral vector (RAdnCD40L) or sCD40L in the CD40-expressing bladder carcinoma T24 cells. The study focuses on data related to immune processing in order to better understand the differential effect of mCD40L versus sCD40L on immune regulatory processes. We further investigated the effects of the different modes of CD40 ligation on immune cell activation and function.

## Results

### Transcriptional profiling to examine the differential effects of membrane-bound CD40L (mCD40L) and soluble CD40L (sCD40L) on CD40-expressing carcinoma cells with a focus on immune function

Previously, we have shown that mCD40L can directly induce apoptosis in CD40-expressing carcinomas, through a mechanism involving sustained activation of the pro-apoptotic JNK pathway and downregulation of the pro-survival PI3K/AKt pathway^11,12^. In contrast, induction of cell death by sCD40L requires inhibition of the protein synthesis machinery^14^.

**Figure 1:**
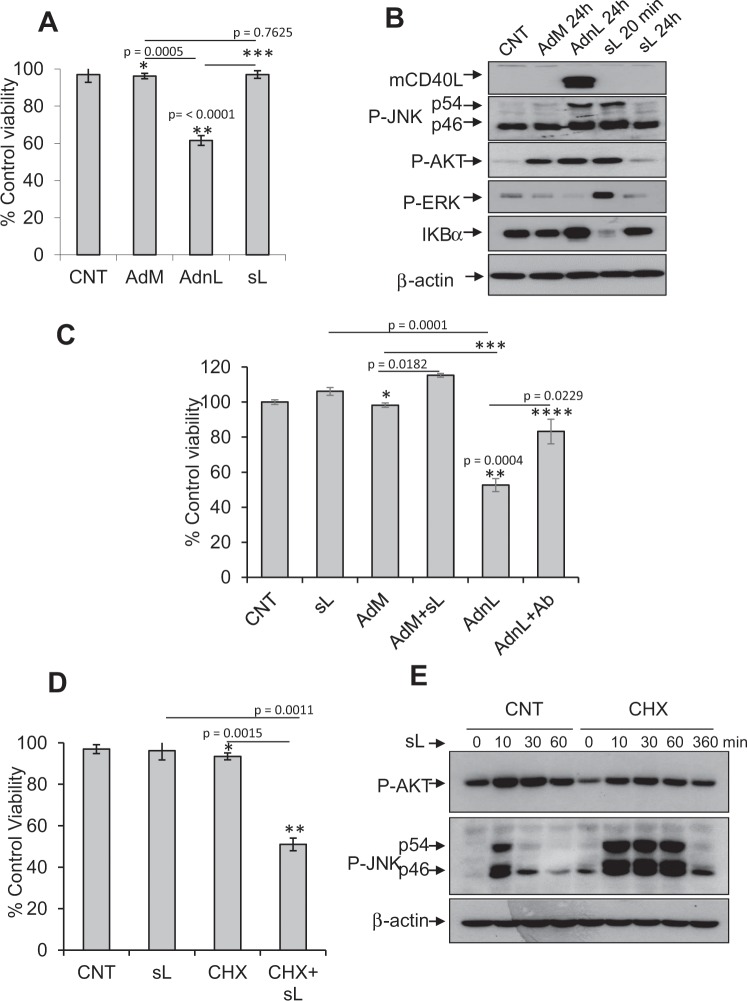
RAdnCD40L but not sCD40L induces cell viability reduction in T24 bladder carcinoma cells. T24 cells were infected with 100 multiplicity of infection (MOI) of either recombinant adenovirus expressing GFP control, RAdMock (AdM) or RAdnCD40L (AdnL) or left untreated as a control or treated with 1 µg/ml of sCD40L (sL). (**A**) Cell viability was assessed 36 hours post-treatment using WST-1 assay. Results represent mean of triplicate samples ± SD. Two-tailed t-test of AdnL/AdM*, AdnL/sL** and AdM/sL***. (**B**) Total cell lysates were probed with anti-phospho -JNK, -AKT and –ERK, anti- β-actin, anti-ikβα and anti-CD40L Abs. (**C**) T24 cells were infected with 100 MOI of either RAdMock (AdM) or RAdnCD40L (AdnL) or left untreated as a control or treated with sL, AdM-infected cells were either treated with sL or left untreated as a control, RAdnCD40L-infected cells were either treated with 5 µg/ml of anti-CD40L neutralizing mAb or left untreated, cells were incubated for 36 hours before cell viability assessment by WST-1 reagent. Results represent mean of triplicate samples ± SD. Two-tailed t-test AdM + sL/sL*, AdM/AdnL**, AdnL/sL*** and AdnL + Ab/AdnL****. (**D**) T24 cells were pre-treated with cycloheximide (CHX) at a concentration of 30 µg/ml for 3 hours before addition of sL (1 µg/ml) and CHX (30 µg/ml). Following 36 hours incubation, cell viability was assessed with results represent mean of triplicate samples ± SD. Two-tailed t-test CHX/CHX + sL* = 0.0015 and CHX + sL/sL** p = 0.0011 (**E)**. Total cell lysates were probed with anti-phospho −JNK, −AKT, and β-actin as a loading control.

In line with our previous work, bladder carcinoma T24 cells transduced with RAdncCD40L for 36 hours exhibited significant reduction in cell viability, while sCD40L (1 µg/ml, 36 h) treatment did not show any cell viability reduction (Fig. 1A) despite full sCD40L biological activity (Fig. 1B) evidenced by phosphorylation of JNK, PI3k/Akt and ERK kinases in addition to IKβα degradation, where sCD40L addition resulted in comparable levels of AKT and JNK phosphorylation to those transduced with RAdnCD40L at 20 min treatment. The ability of CD40L monoclonal neutralizing antibody to restore cell viability in RAdnCD40L-infected cells further attributes cell death to mCD40L expression (Fig. 1C). Furthermore, the inability of sCD40L to induce any cell death in AdM-infected cells also excludes any synergy between the adenoviral vector proteins and CD40-CD40L interaction in mCD40L-induced cell death (Fig. 1C).

Inhibition of protein synthesis machinery appeared to be a prerequisite for T24 cell death induced by sCD40L (Fig. 1D), since pre-treatment of T24 cells with cycloheximide (CHX) at a concentration of 30 µg/ml for 3 hours followed by sCD40L (1 µg/ml) and CHX (30 µg/ml) treatment for 36 hours resulted in significantly reduced cell viability compared to either single treatment alone. Co-culturing T24 cells with CHX and sCD40L resulted in strong sustained JNK phosphorylation (Fig. 1E) at 10, 30 and 60 minutes compared to transient JNK phosphorylation at 10 minutes by sCD40L, while CHX treatment alone did not induce JNK phosphorylation.

**Figure 2:**
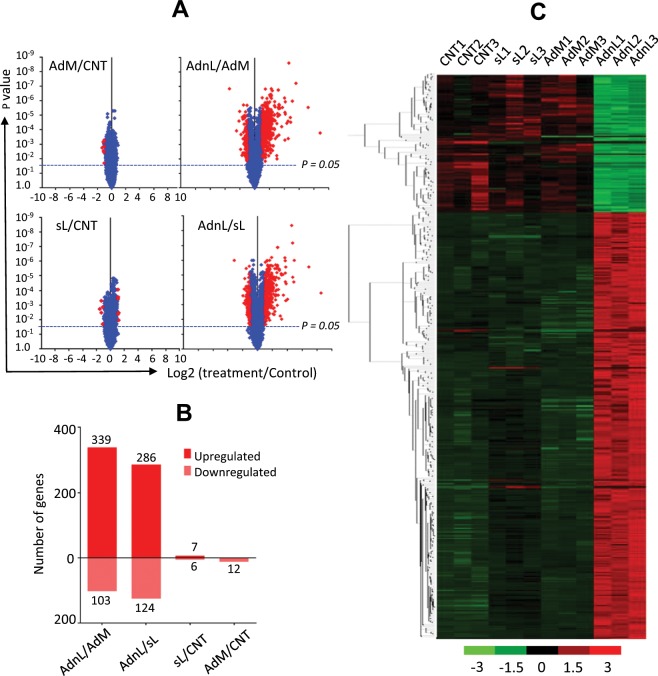
Microarray gene expression analysis. Gene expression levels were analysed by the Affymetrix Expression Console software with the RMA-sketch workflow as a default settings. **(A)** Normalized microarray data (Treatment vs Control) were visualised by GraphPad 7 Volcano blot analysis with blue-coloured squares representing genes with either P value > 0.05 and/or expression Fc < 2 compared to control treatment, red-coloured squares represent those with ≥2 Fc and P value ≤ 0.05. **(B)** Chart indicating the number of significantly altered genes (≥2 Fc with P value < 0.05) in RAdnCD40L-infected cells compared to RAdMock cells (AdnL/AdM), RAdnCD40L-infected cells compared to sCD40L (AdnL /sL), RAdMock-infected cells compared to untreated cells (AdM/CNT) and sCD40L-treated compared to untreated cells (sL/CNT). **(C)** Significantly altered genes (≥2 Fc and P value ≤ 0.05) were visualised by hierarchical clustering heat map highlighting the differential expression levels between AdnL compared to AdM, sCD40L (sL: 1 µg/ml) treated and untreated control (CNT) cells. Gene expression values were row-scaled to have mean value of 0 and standard deviation of 1.

To fully appreciate the differential effects of mCD40L and sCD40L on the outcome of CD40 ligation, we examined the transcriptional profiling of the bladder carcinoma T24 cells in response to CD40 ligation by either mCD40L or sCD40L. Gene expression data from three biological experiments for each treatment were analysed with limma software. Differentially expressed genes were identified with the criteria of absolute fold change greater than 2 and p value less than 0.05 (Fig. 2A). A total 410 genes were found to be differentially expressed by mCD40L compared to sCD40L treatment (RAdnCD40L/sL) (Fig. 2B), of which 286 genes were upregulated, while 124 genes were downregulated. On comparison of sCD40L to untreated control cells (sL/CNT), 12 genes were differentially expressed. Whilst, only 12 genes showed reduced expression in RAdMock infected cells compared to untransduced cells (AdM/CNT). Hierarchical clustering analysis of those transcripts with absolute fold change (Fc) ≥2 and p value < 0.05 induced by mCD40L compared to sCD40L treatment indicates clusters of functionally annotated gene sets (Fig. 2C).

**Figure 3:**
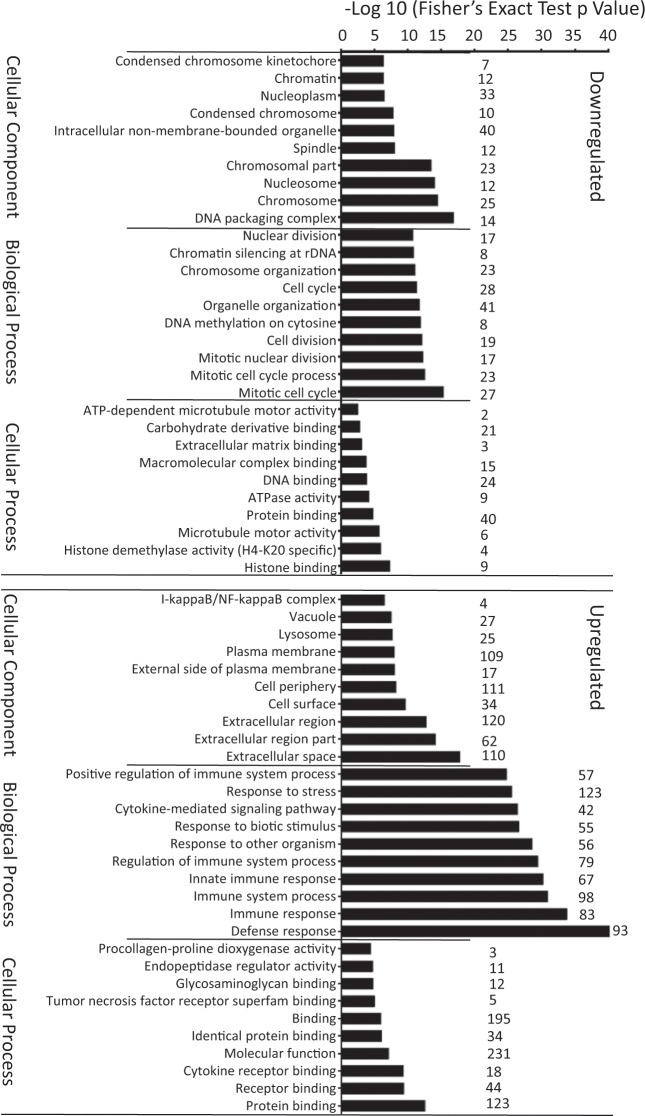
Gene annotation enrichment of differentially altered transcripts in RAdnCD40L-transfected T24 cells compared to sCD40L-treated cells. Transcripts with significant alteration (≥2 Fc and P value ≤ 0.05) in RAdnCD40L-transfected T24 cells compared to sCD40L-treated cells were analyzed by the online-available string software http://string-db.org/ for pathways enrichment. The top 10 significant pathways in either upregulated or downregulated transcripts with the number of genes in each pathway are highlighted by Fisher’s exact test p value analysis.

To assist interpretation, gene ontology enrichment (GO) analysis of those transcripts either upregulated (286 genes) or downregulated (124 genes) by mCD40L compared to sCD40L treatment was performed utilising the web-available protein-protein interaction networks String database (https://string-db.org). The top 10 functional pathways of the upregulated genes were predominantly implicated in immune-related functions, whilst those of downregulated genes appeared to be critical for cell cycle and proliferation (Fig. 3 and Supplementary Data 1 and 2 respectively).

### MCD40L induces higher expression of transcripts involved in immune responses compared to sCD40L in CD40-expressing carcinoma cells

**Figure 4:**
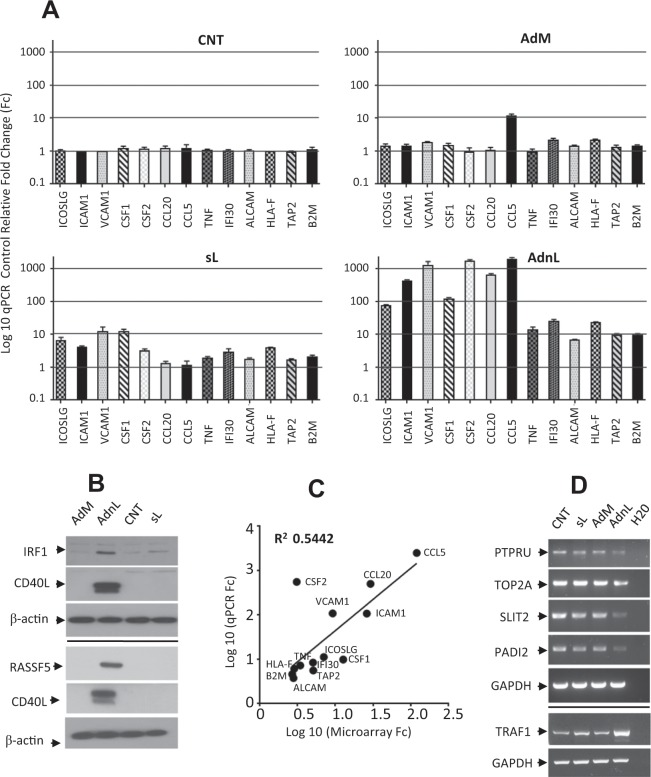
Microarray results validation. T24 cells were infected with 50 MOI of either RAdnCD40L (AdnL) or RAdMock (AdM) or treated with sCD40L (sL) at a concentration of 1 µg/ ml for 24 hours or left untreated as a control (CNT). (**A**) Expression levels of the indicated transcripts were examined utilising cDNA synthesised from total RNA isolated from cells by qRT-PCR technique. Results represent the mean of three replicate pairs ± SD. (**B**) 50 µg of total cell lysates were examined by western blot analysis for IRF1, RASSF5 and CD40L expression, β-actin was examined to ensure equal inputs. (**C**) Correlation analysis of qPCR and microarray results of the qPCR-selected transcripts. (**D**) RT-PCR analysis of PTPRU, TOP2A, SLIT2, PADI2 and TRAF1 transcripts were also examined utilising cDNA as a template. GAPDH was examined to ensure equal cDNA input across different treatment.

Results from gene ontology enrichment analysis suggest that mCD40L upregulates the immune-activation machinery. Therefore, we validated the expression of a selection of transcripts (Table 1) including genes with critical roles in antigen processing and presentation, cell adhesion molecules, cytokines, cytokine receptors, chemokines and co-stimulatory molecules. Thus, T24 cells were infected with RAdnCD40L or treated with sCD40L and gene expression validation was carried out at the transcriptional level (Fig. 4A) by qRT-PCR techniques and at the protein level (Fig. 4B) by western blot analysis, with significant correlation with the corresponding microarray data (Fig. 4C). Furthermore, some of the genes negatively regulated by mCD40L (Table 1), including peptidyl arginine deiminase, type II (PADI2), the most negatively affected transcript (Fc, 8.82; P = 3.07 × 10^−11^), topoisomerase (DNA) II alpha (TOP2A), Slit guidance ligand 2 (SLIT2) and protein tyrosine phosphatase, receptor type, U (PTPRU) were also validated by RT-PCR. RT-PCR analysis of PADI2, TOP2A, SLIT2 and PTPRU indicate reduced expression corresponding to the microarray data (Fig. 4D).

**Table 1:** Expression validation of a selection of genes differentially expressed by mCD40L compared to sCD40L (RAdnCD40L/sL).

Gene Description	ID	RefSeq	P-value	Fc
Chemokine (C-C motif) ligand 5	CCL5	NM_001278736	8.45E-14	120.35
Chemokine (C-C motif) ligand 20	CCL20	NM_001130046	1.01E-12	29.37
Intercellular adhesion molecule 1	ICAM1	NM_000201	1.13E-12	26.2
Colony stimulating factor 1 (macrophage)	CSF1	NM_000757	9.65E-12	12.73
Vascular cell adhesion molecule 1	VCAM1	NM_001078	8.05E-08	9.23
Inducible T-cell co-stimulator ligand	ICOSLG	NM_001283050	5.66E-09	7.06
Transporter 2, ATP-binding cassette	TAP2	NM_000544	1.71E-09	5.12
Interferon, gamma-inducible protein 30	IFI30	NM_006332	2.67E-09	5.08
Tumor necrosis factor	TNF	NM_000594	1.99E-08	3.43
Colony stimulating factor 2 (granulocyte-macrophage)	CSF2	NM_000758	7.05E-08	3.08
Major histocompatibility complex, class I, F	HLA-F	NM_001098478	2.00E-07	2.86
Activated leukocyte cell adhesion molecule	ALCAM	NM_001243280	1.66E-07	2.8
Beta-2-microglobulin	B2M	NM_004048	1.54E-06	2.69
Slit guidance ligand 2	SLIT2	NM_001289135	3.08E-07	−2.77
Topoisomerase (DNA) II alpha	TOP2A	NM_001067	9.76E-08	−2.79
Protein tyrosine phosphatase, receptor type, U	PTPRU	NM_001195001	9.26E-09	−3.88
Peptidyl arginine deiminase, type II	PADI2	NM_007365	3.07E-11	−8.82

In line with our previous findings, indicating that mCD40L induces cell death in CD40 positive carcinomas by influencing the balance between apoptotic and survival signals, through posttranscriptional stabilization of TNFR-associated factor 3 (TRAF3) and destabilization of TRAF6, with no evidence of mCD40L inducing transcriptional activity in regards to TRAF3 and TRAF6^12^, no changes in the transcriptional levels of TRAF2, TRAF3, TRAF5 and TRAF6 have been detected, however TRAF1 was upregulated in RAdnCD40L compared to sCD40L (AdnL/sL; FC 30.5: p = 2.8 × 10^13^) and confirmed as well by RT-PCR analysis (Fig. 4D). Furthermore, we have also shown previously that mCD40L-induced NORE1A (RASSF5) expression contributes to mCD40L-induced cell death in JNK-independent mechanism^13^. Indeed RAdnCD40L but not sCD40L induced RASSF5 expression at the transcriptional level (AdnL/sL; FC 9.6: p = 1.62 × 10^−12^), that has been confirmed by western blot analysis (Fig. 4B).

### MCD40L enhances dendritic cell (DC) maturation and activation

The importance of dendritic cells (DC) as professional antigen presenting cells (APC) stimulating cytotoxic T lymphocytes is well-established^15^. This requires DC maturation and activation. Following antigen uptake, DC undergo phenotypic alteration and express higher levels of co-stimulatory molecules including CD54, CD83, CD80 and CD86^16^. Activation of CD40 on DC surface induces functional maturation and enhances the capacity of DC to induce T cell proliferation and secretion of cytokines including IL-1, IL-6, IL-8, IL10, IL12 and TNF-α^17–19^.

Our finding that mCD40L significantly up-regulates expression of several transcripts involved in immune responses in CD40-expressing carcinoma cells promoted us to investigate the effects of mCD40L compared with sCD40L on DC maturation and activation. Thus, immature DC were generated from CD14 + monocytes isolated from peripheral blood of at least 5 different healthy donors by culturing with IL4 + GM-CSF for 5 days. Immature DC were then co-cultured with the CD40- pancreatic cell line CFPAC-1 cells either transduced with RAdnCD40L, or treated with sCD40L or with maturation cocktail (MC: IL-1β, 25 ng/mL, TNFα, 50 ng/mL, IFNγ, 1000 U/mL, IL-6, 1000 U/mL, PGE2, 10^−6^ M, LPS, 100 µg/mL) as a positive control. After 24 hours, DC were retrieved from the co-cultures or left for a further 24 hours. Retrieved DC were examined for cell surface maturation and activation molecules including CD40, CD83, HLA-DR, CD54 and CD86 by FACS analysis. IL-12 and IL-10 in co-culture media samples collected at 24 and 48 hours were also assayed by ELISA.

**Figure 5:**
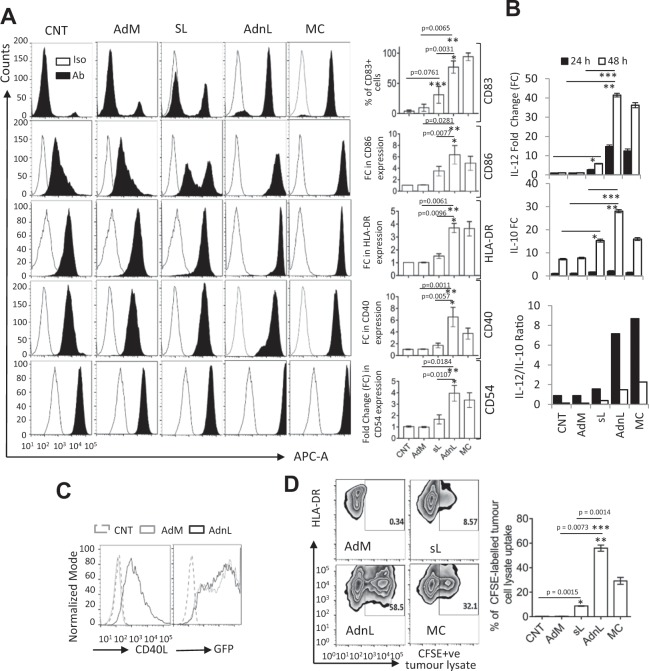
DC maturation and activation by mCD40L. Immature DC (iDC) were co-cultured with CFPAC-1 cell pre-transduced with 50 MOI RAdMock (AdM) or RAdnCD40L (AdnL) or treated with sCD40L (sL; 1 µg/ml) or the maturation cocktail (MC) as a positive control. Activated DC were retrieved from the co-cultures after 24 h for FACS analysis or left for a further 24 h for culture media sampling. **(A)** Expression of CD40, CD83, HLA-DR, CD54 and CD86 were examined by FACS analysis on DC retrieved after 24 hours from different co-cultures. Matching isotype antibodies (Iso) staining were also conducted to ensure the specificity of the utilised antibodies (Abs) in detecting target expression. Results represent the mean of three biological experiments ± SD. Two-tailed t-test analysis of AdL/sL*, AdnL/AdM** and sL/CNT***. (**B**) IL-12 and IL-10 were quantified by ELISA within samples collected from different co-cultures at 24 and 48 hours points. Results mean of three biological experiments ± SD. Two-tailed t-test analysis of sL/CNT* (IL-12; 24 h, p = 0.0032; 48 h, p = 0.0046: IL-10; 24 h, p = 0.0.0318; 48 h p = 0.0059), AdnL/AdM** (IL-12; 24 h, p = 0.0014; 48 h, p = 0.0016: IL-10; 24 h p = 0.0.0051; 48 h p = 0.0049) and AdnL/sL*** (IL-12; 24 h p = 0.0037; 48 h p = 0.0017: IL-10; 24 h p = 0.0.0037; 48 h p = 0.0016). **(C)** Activated DC pulsed with CFSE-labelled necrotic CFPAC-1 cells at 1:1 ratio for 1 h at 37 °C, were analyzed by FACS analysis for CFSE uptake. Results represent the mean of three biological experiments ± SD. Two-tailed t-test analysis comparing sL/CNT*, AdnL/AdM** and AdnL/sL***. (**D**) mCD40L expression was examined in AdnL-transduced CFPAC-1 cells in addition to GFP to ensure equal adenoviral infection compared to AdM control.

As shown in Fig. 5, DC co-cultured with CFPAC-1 cells expressing mCD40L exhibited higher expression levels maturation and activation (Fig. 5A) with higher detection levels of IL-12 and IL-10 in samples collected at 24 and 48 hours points (Fig. 5B) compared to sCD40L. However, mCD40L-induced maturation and activation of DC were comparable to those induced by MC, indicating enhanced DC activation by mCD40L over sCD40L. This was clearly attributable to mCD40L expression since RAdMock-transduced or untransduced CFPAC-1 cells failed to induce DC maturation. To confirm that CFPAC-1 cells were transduced with equal amounts of RAdMock and RAdnCD40L, the expression levels of mCD40L and GFP were examined by FACS analysis. As shown in Fig. 5C, similar levels of GFP expression were detected in both RAdMock and RAdnCD40L-infected cells, with mCD40L only expressed in RAdnCD40L-infected cells.

To examine the tumour antigens uptake capacity of activated DC generated across different treatments, activated DC were pulsed with equal amount of CFSE-labelled necrotic CFPAC-1 cells at 1:1 ratio for 1 h at 37 °C, followed by FACS analysis for CFSE uptake. As shown in Fig. 5D mCD40L-activated DC exhibited significantly higher CFSE-labelling compared with those activated by MC, whilst sCD40L-activated DC exhibited only limited antigen-uptake. Taken together, these results indicate that mCD40L induces effective functional DC activation significantly more than sCD40L.

### mCD40L-activated DC enhance T-cell proliferation and cytotoxic T lymphocyte (CTL) response compared with sCD40L

To assess the ability of mCD40L-activated DC to stimulate T cell proliferation, retrieved DC from different treatment were pulsed with CFPAC-1 cell lysate at a ratio of 1:5 (DC: tumour cell equivalent) for 24 hours. Tumour-loaded DC were incubated with CFSE-labelled autologous CD3+ T cells at a responder to-stimulator (R:S) T-cell/DC ratio of 10:1. To evaluate the T cells response on day 5, we analyzed CD3+ T cells for CD8+ T cells by gating CFSE negative or low stained CD3 + CD8+ T cells utilizing anti-CD3-pacific blue and anti-CD8-AlexaFluor 700 respectively, based on CFSE dilution^20^ as a result of T cell proliferation compared with CFSE strong staining of unproliferated T cells. A minimum of 100,000 CD3 + cells were analyzed for CD8 + populations by flow cytometry. The results were expressed as the percentage of CFSE low or negative cells relative to CD3 + CD8+ T cells.

**Figure 6:**
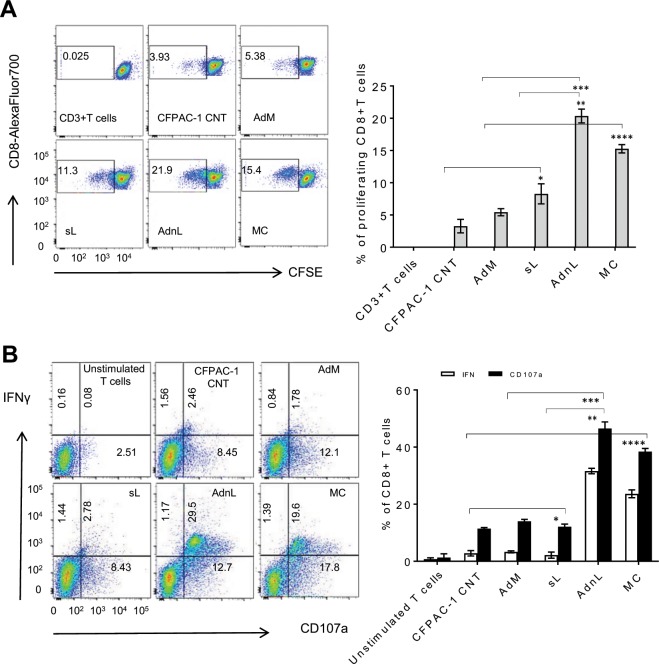
T-cell proliferation and cytotoxic response to mCD40L-activated DC compared with sCD40L. DC co-cultured for 24 hours with CFPAC-1 cells (CFPAC-1 CNT) alone or CFPAC-1 cells pre-transduced with 50 MOI RAdMock (AdM) or RAdnCD40L (AdnL) or treated with sCD40L (sL; 1 µg/ml) or the MC were retrieved and loaded with CFPAC-1 tumour lysate. (**A**) CFSE-labelled autologus CD3+ T cells were incubated with tumour cell lysate-loaded DC at a responder to-stimulator (R:S) T-cell/DC ratio of 10:1 for 5 days or cultured alone as a negative control. Retrieved CD3+ T cells were examined for CD8+ T cells by gating CD3 + CD8+ T cells population utilizing anti-CD3-Pacific blue and anti-CD8-Alexa Fluor 700. CD8+ T cells were selected by gating CD3 and CD8 double stained cells with negative or low CFSE. The results were expressed as the percentage of CFSE negative or low cells with Pacific blue and Alexa Fluor 700 positive staining and represent the mean of three biological experiments ± SD. Two-tailed t-test analysis comparing different treatments including sL/CNT*(p = 0.1515, p = 0.0334), AdnL/sL**(p = 0.0059, p = 0.0148), AdnL/AdM*** (p = 0.0083,p = 0.0132) and MC/CNT****(p = 0.0091, p = 0.0024). (**B)** *In vitro* expanded T cells obtained from co-culture with DC loaded with tumour lysate for 7 days were stimulated for 5 hours with irradiated CFPAC-1 cell lysate-loaded autologous PBMCs. Unstimulated CD3+ T cells were used as a negative control (unstimulated T cells). Protein transport inhibitor, GolgiStop and anti-CD107a PE Ab were added 1 hours after stimulation. Cells were stained with anti-CD3-Pacific blue, anti-CD8-AlexaFluor 700 and anti-IFN-γ APC. Anti-CD3, -CD8 positive stained were gated by flow cytometery and analyzed for CD1017a and IFN-γ positive staining cells. Results represent the mean of three biological experiments ± SD. Two-tailed t-test analysis comparing different treatments including sL/CNT* (p = 0.3671, p = 0.5739), AdnL/sL** (p = 0.0043, p = 0.0025), AdnL/AdM*** (p = 0.0008, p = 0.0068) and MC/CNT**** (p = 0.0066, p = 0.0026) for IFN-γ and CD1017a positive cells respectively.

As shown in Fig. 6A, incubation of CFPAC-1 tumour lysate-loaded mCD40L-activated DC loaded with autologus CD3+ T cells stimulated higher percentage of CD3+ T cell proliferation into CD8+ T cells compared with MC-activated DC, however significantly higher than those stimulated with sCD40L-activated DC or DC isolated from RAdMock-transduced CFPAC-1 co-culture, or untreated CD3+ T cells. To evaluate the functional cytotoxic activity of these *in vitro* expanded T cells, we examined CD107a degranulation and intracellular IFN-γ production. The importance of CD107a degranulation for immediate lytic function by T lymphocytes is well-recognized^21^. Thus, proliferated T cells in response to CFPAC-1-tumour lysate-loaded activated DC generated across different treatments were stimulated with irradiated cell lysate-loaded autologous PBMC. GolgiStop and anti-CD107a PE Ab were added 1 hour after stimulation and incubated for 5 hours. Retrieved T cells were stained with anti-CD3-Pacific blue, anti-CD4-FITC and anti-CD8-AlexaFluor 700. Following fixation and permeabilization with Cytofix/Cytoperm solution, cells were stained with anti-IFN-γ APC and analysed for CD3+ CD8+ CD4− cells with positive CD107a and IFN-γ staining.

As shown in Fig. 6B, T cells expanded via mCD40L-activated DC exhibited a higher percentage of CD107a degranulation and IFN-γ production compared to sCD40L, indicating that mCD40L-activated DC are functionally active and are capable of inducing increased T cell proliferation and cytotoxic response compared to sCD40L-activated DC.

## Discussion

In immune cells, CD40-CD40L interaction is critical in orchestrating immune responses including DC maturation and activation with ability to initiate T-cell responses^22^.

However, in CD40 + carcinomas, CD40 ligation via mCD40L but not sCD40L has been reported to induce cell cycle arrest and apoptosis^11–14^, through a mechanism involves constitutive activation of the pro-apoptotic JNK pathway and downregulation of PI3K^11,12^, a known anti-apoptotic effector and regulator of gene expression^23^.

In line with that, mCD40L but not sCD40L induced cell death in the CD40+ T24 cells. However, sCD40L-induced cell death required protein synthesis inhibition by CHX, suggesting that sCD40L induces potent survival signals capable of suppressing its pro-apoptotic effects. CHX treatment appears not only shifting the balance between sCD40L-induced survival and pro-apoptotic signals by disrupting the survival signals but also by enhancing the pro-apoptotic JNK activation by prolonging its activity, a critical requirement in mCD40L-induced cell death.

In view of broadly understanding the differential effects of CD40 ligation by mCD40L versus sCD40L, we compared the T24 cells transcriptome following CD40 ligation by sCD40L (24 h) and mCD40L (24 h), and by subjecting our microarray data to absolute fold change (Fc) ≥ 2 and p value < 0.05, we ensured that only significant transcriptional changes are selected. Furthermore, by utilising the gene ontology enrichment (GO) analysis we were able to categorise the differentially altered transcripts based on the significance of their functional pathways. Where, the top 10 significant functional pathways of those upregulated or downregulated by mCD40L compared to sCD40L were identified. Upregulated transcripts were predominantly implicated in immuno-related functions, while those downregulated were pivotal for cell cycle and proliferation (Fig. 3). Interestingly, mCD40L not only upregulated genes enhancing immunostimulatory function but also downregulated those negatively impacting this process, such as SLIT2, which is involved in inhibition of chemotaxis and adhesion of monocytes to activated human endothelial cells and to immobilized ICAM-1 and VCAM-1 cells^24^.

To examine the transcriptional activity underlying mCD40L-induced cell death, we examined transcriptional changes of TNFR-associated factors (TRAFs) including TRAF1, 2,3,5 and 6, as CD40 lacks any intrinsic kinase activity and only rely on recruiting TRAFs to its cytoplasmic domain in order to convoy its signals^25–27^.

Interestingly, only TRAF1 among other TRAFs molecules including TRAF2, TRAF3, TRAF5 and TRAF6 was upregulated by mCD40L (AdnL/sL: Fc 30.5: p = 2.8 × 10^13^), reconfirming our previous observation that mCD40L induced JNK activation and PI3K down-regulation is mediated by post-transcriptional stabilization and destabilization of TRAF3 and TRAF6 respectively^11,12^. RASSF5 (NORE1A), another important molecule that mediates mCD40L-induced cell death in JNK-independent mechanism^13^ also was upregulated by mCD40L but not sCD40L (AdnL/sL; FC 9.6: p = 1.62 × 10^−12^). Taken together, these data further reassure our microarray results.

CD40 ligation on surface of immature DC promotes DC maturation and activation demonstrated by cytokine production, induction of costimulatory molecules on their surface, and cross-presentation of antigen^15,18^. However, for induction of higher level of Th1-polarizing cytokine IL-12 secretion by DC, a combination of CD40L and IFN-γ are required^28–30^. Indeed, DC stimulation with sCD40L alone appeared to be less efficient, demonstrated by low level of IL-12 secretion and reduced antigen-uptake capacity by sCD40L-activated DC. In contrast, mCD40L-activated DC not only enhanced the antigen-uptake but also induced higher levels of IL-12 secretion, albeit higher IL-10 was also observeed, however the IL-12:IL-10 ratio remains significant. In agreement with our results, CD40L-expressing DCs was reported to induce higher IL-12 expression compared to TNF-α activated DC, moreover addition of IL-10 did not compromise CD40L-induced DC activation^31^.

Efficient DC maturation and activation is a key checkpoint in priming T cells, with the nature of DC maturation influencing IL-12 induction^32^, a critical Th1 polarising cytokine^33^. DC maturation by CD40L is known not only to induces IL-12 production^28^, but also for its capacity to initiate Th1-type responses against tumours^34,35^.

Indeed, autologus CD3+ T cells strongly responded to CFPAC-1 tumour lysate-loaded mCD40L-activated autologus DC with higher percentage of proliferation into CD8+ T cells compared to sCD40L.

Furthermore, the positive correlation between levels of T cell proliferation with levels of CFPAC-1 tumour uptake by activated DC (Fig. 5D) across different treatments, suggests CD3+ T cells proliferated in response to CFPAC-1 tumour antigens, particularly that tumour-unloaded activated DC are known to be incapable of electing T cell response^36^.

In line with that, incubation of irradiated CFPAC-1 tumour-loaded autologous PBMCs with CFPAC-1 tumour specific CD3 + CD8+ T cells in response to CFPAC-1-tumour lysate-loaded mCD40L- activated DC stimulated CD3 + CD8+ T cells cytotoxic response and resulted in higher percentage of CD3 + CD8+ T cells with positive staining for IFN-γ and CD107a compared to those CFPAC-1 tumour specific CD3 + CD8+ T cells generated in response to CFPAC-1-tumour lysate-loaded DC activated through other treatments including sCD40L and MC. Where, CFPAC-1 tumour lysate-loaded RAdMock-activated autologus DC or control CFPAC-1 tumour lysate-loaded CFPAC-1 retrieved-DC were not efficiently matured to elicit T cell response indicating that our observed T cell response is a mCD40L-dependant. Furthermore, in our model we selected the CD40- CFPAC-1 cells to deliver mCD40L rather than a CD40 + cell line to avoid any potential confounding effects due to apoptotic induction by RAdnCD40L.

The mechanism by which mCD40L enhances immune responses is not yet fully understood, however it could be due to the signal potency delivered by mCD40L, given that mCD40L transduces prolonged signals compared to sCD40L in CD40 + carcinomas^12^. Furthermore, our microarray analysis revealed upregulation of STAT5a transcription by mCD40L compared to sCD40L (AdnL/sL; Fc 2.9, p = 6.1 × 10^−8^), STAT5a is key regulator of inflammatory cytokines gene expression including TNFα, interferon-γ (IFN-γ), and interleukin-6 (IL-6)^28^. Inhibition of JAK3 pathway that mediates STAT5s expression in CD40-mediated DC maturation resulted in tolerogenic DC conversion^37^.

Thus, mCD40L expression in transduced tumour cells may induce apoptosis in CD40-expressing carcinomas, liberating tumour associated antigens for uptake by tumour-infiltrating dendritic cells. At the same time, mCD40L expression on tumour cells may directly stimulate these DC for enhanced antigen presentation to CD8+ T-cells, promoting their proliferation and tumour-specific cytotoxic responses. However, we cannot exclude possible role of CD4+ T cells for CD8+ T cell activation under this experimental condition, as we have not examined the purified CD8+ T cells response.

**Figure 7:**
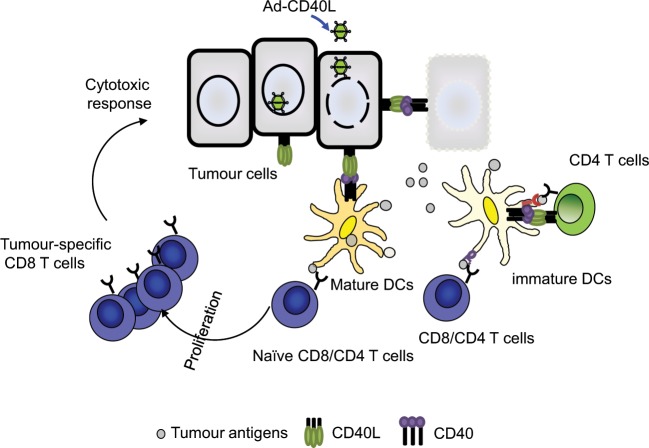
Multiple mechanisms of action of mCD40L immunostimulatory therapy. mCD40L expression in transduced tumour cells may induce apoptosis in CD40-expressing carcinomas, liberating tumour associated antigens for uptake by tumour-infiltrating dendritic cells. At the same time, mCD40L expression on tumour cells may directly stimulate these DC for enhanced antigen presentation toward CD4 + and CD8+ T-cells, promoting their proliferation and tumour-specific cytotoxic responses.

Collectively, these finding suggest that mCD40L could be harnessed as a potent immunostimulatory cancer therapy given its ability to stimulate different facets of the immune response together with its ability to directly induce cell death in CD40-expressing carcinomas, which itself may release tumour specific antigens into the tumour microenvironment to further enhance anti-tumour immune responses (Fig. 7).

## Materials and Methods

### Cells and maintenance

Bladder carcinoma cell line T24 cells and the pancreatic cell line CFPAC-1 cells (originally obtained from the ATCC, cat no ATCC HTB-4 and ATCC CRL-1918 respectively and routinely examined for mycoplasma by PCR every 6 month) were maintained in either RPMI 1640 or DMEM supplemented with 2 mM glutamine, 10% FBS. Peripheral blood mononuclear cells (PBMCs) were isolated from heparinized healthy donors’ blood by using density gradient centrifugation on lymphoprep (Axis-Shield PoC AS, Oslo, Norway) according to the manufacturer’s instructions. Isolated PBMCs were washed twice with phosphate-buffered saline (PBS) then resuspended in RPMI-1640 media. CD14 + monocytes and CD3+ T cells were isolated from fresh PBMCs by positive selection using anti-CD14 and anti-CD3-coated magnetic beads and magnetic columns according to the manufacturer’s instructions (Miltenyi Biotec, Germany). The purity of monocytes and T cells was routinely >95%, as assessed by flow cytometry using anti-CD14 and anti-CD3 antibodies respectively.

Immature dendritic cells (iDC) were generated by culturing CD14 + monocytes in complete RPMI-1640 medium supplemented with 10% FBS (Sigma-Aldrich), recombinant human IL-4 cytokine (800 U/ml) and granulocyte/monocyte-colony stimulating factor (GM-CSF; 800 U/ml) (PeproTech, London, UK). At day 3, cultured cells were topped up with medium containing IL-4 (400 U/ml) and GM-CSF (400 U/ml). At day 5, cells were harvested and analyzed for the expression of CD1a, CD14 and CD83 markers.

### Recombinant adenovirus vectors and cell infection

The replication-deficient E1, E3-deleted recombinant adenoviruses expressing either membrane-bound, noncleavable CD40L (RAdnCD40L), or GFP control (RAdMock) were constructed using methods as previously described^38^. Viruses were purified by caesium chloride banding and dialyzed against a buffer containing 10 mM Tris-HCl (pH 8.0), 2 nM MgCl, and 5% sucrose. Virus titers were determined using the 50% tissue culture-infective dose method, based on the development of cytopathic effects in HEK 293 cells using serial dilutions to estimate adenovirus stock titer. Cells were infected in 10% FBS DMEM with the appropriate multiplicity of infection (MOI) for 2 hours at 37 °C. The resistance of the membrane-bound CD40L mutant expressed and delivered by RAdnCD40L to cleavage from the cell membrane was confirmed and described previously^12^.

### WST-1 cell viability assay

Cells were plated in 96-well plates at 4000 cell/100 μl/ well and incubated at 37 °C. Cell viability was assessed by adding WST-1 reagent (Roche) to the culture medium at 1:10 dilution. Cells were incubated at 37 °C and the optical density was measured by microplate ELISA reader at λ450 every 2 hours to a maximum of 6 hours. The amount of the formazan formed directly correlates to the number of metabolically active cells.

### Treatment of cells and RNA isolation and extraction for microarray analysis

T24 cells were infected with 50 MOI RAdMock or RAdnCD40L or left uninfected for 24 hours or treated with sCD40L at a final concentration of 1 µg/ml (PeproTech, London, UK) for 24 hours, total RNA was extracted from cells using the EZ-RNA total isolation kit (Biological Industries, Israel) according to the manufacturer’s instructions with RNA integrity and quality confirmed by Agilent Bioanalyser.

### cDNA probe synthesis

200 ng of total RNA was converted to cDNA using a WT Expression kit (Ambion, Cambridge, MA, USA) according to the manufacturer’s procedures. Resultant cDNA was fragmented and labelled with biotin using an Affymetrix WT Terminal Labelling kit (Affymetrix, Santa Clara, CA, USA).

### Microarray hybridisation analysis

Biotin-labelled cDNA was hybridised to Affymetrix Human Gene 1.0 ST v1 arrays. Arrays were then washed and stained on an Affymetrix FS400 fluidics station, followed by scanning utilising an Agilent G2500A GeneArray scanner according to the manufacturer’s instructions. GCOS software (Affymetrix) was used for instrument control and data acquisition. Gene level analysis was performed using the Affymetrix Expression Console software with the default settings of the RMA-sketch workflow^39^. Differentially expressed probe sets were identified via the linear models and empirical Bayes method^40^. Raw microarray data were submitted to the public repository Gene Expression omnibus (GEO) (GEO Accession: GSE98891).

### RT-PCR analysis

Using the RETROscript RNase reverse transcription kit (Ambion Europe, Huntingdon, UK) according to the manufacturer’s instructions, cDNA was synthesised from total RNA extracted by EZ-RNA total isolation kit (Biological Industries, Israel). RT-PCR was performed using PLATINUM Taq DNA polymerase (Invitrogen) utilizing the human PTPRU-specific primers: forward 5′-ggagaggctactctgcccg-3′ and reverse 5′-ggtgaccgagttcagcgtctg-3′, the human TOP2A-specific primers: forward 5′-ggcaccgagaagacacctcct-3′ and reverse 5′-catctggaaccttttgctgggc-3′, the human SLIT2-specific primers: forward 5′-ctcgttgtgctggtcctggag-3′and reverse 5′-gttcaggtcctgggcacagaag-3′, the human PADI2-specific primers: forward 5′-gaactgtgaccgagagacaccc-3′ and reverse 5′-ggaacaggtaattatccttcatgcagc-3′ and the human TRAF1: forward 5′-gttcatgaaacagtggaaggc-3′ and reverse 5′-ggagaagaggctgacggtcct-3′. The amount of cDNA template used for the RT-PCR was adjusted on the basis of amplification of human GAPDH utilising the human GAPDH-specific primers: forward 5′-cctccaaaatcaagtggggcg-3′ and reverse 5′-accaccaggtgctcagtgtag-3′ to ensure equal inputs between different samples.

### QRT-PCR measurement of cellular gene expression

The following TaqMan gene expression assays for B2M (Hs00984230_m1), CCL20 (Hs01011368_m1), CCL5 (Hs00982282_m1), CSF1 (Hs00174164_m1), CSF2 (Hs99999044_m1), HLA-F (Hs01587840_m1), ICOSLG (Hs00391287_m1), IFI30 (Hs00173838_m1), TAP2 (Hs00241060_m1), TNF (Hs00174128_m1), VCAM1 (Hs00365486_m1), ALCAM (Hs00233455_m1) and ICAM1 (Hs00164932_m1) were all selected from the Applied Biosystems website (http://www.appliedbiosystems.com) together with 18 S, GAPDH and HPRT1 rRNAs as a standard control, assembled onto a microfluidics card (Applied Biosystems) and analysed using an ABI Prism 7900HT Sequence Detection System. Individual 50 µl qRT-PCRs were performed for selected candidates. The relative quantity (RQ) of RNA for each gene across the different treatments within the experiment was calculated as described previously^41^. The mean of the RQs from the three replicate pairs was calculated and used in further analysis.

### Western blot analysis and antibodies

Antibodies (Abs) against CD40L and IRF1 were from Santa Cruz Biotechnology. Phosphospecific JNK, AKT and ERK antibodies, IκBα antibody and RASSF5 monoclonal antibody were all from Cell Signaling Technology. Monoclonal mouse anti-β-actin was from Sigma, UK. For immunoblotting, 10-50 μg protein was separated by SDSPAGE and transferred onto nitrocellulose membranes followed by blocking with 10% non-fat milk dissolved in TBS supplemented with 0.1% Tween 20. After three washes with TBS supplemented with 0.1% Tween 20, nitrocellulose membranes were incubated overnight at 4 °C with the primary Abs and for 1 hour at room temperature with the appropriate secondary Abs followed by enhanced chemiluminescence (Amersham Biosciences, Piscataway, NJ).

### Immature DC activation

CFPAC-1 cells were transduced with either 100 MOI RAdMock or RAdnCD40L or left un-transduced as a negative control for 24 hours followed by co-culturing with iDC at a ratio of 1:2 cells to iDC. Uninfected CFPAC-1 and iDC co-culture control were either treated with soluble CD40L (1 µg/ml) or MC (IL-1β: 25 ng/mL (Enzo Life sciences, UK), TNFα: 50 ng/mL (Enzo life sciences, UK), IFNγ: 1000 U/mL (R&D Systems, Inc, USA), IL-6: 1000 U/mL (R&D Systems, Inc, USA), PGE2: 10^−6^ M (Sigma-Aldrich, Ltd, Dorset, England, UK), LPS: 100 µg/mL (Enzo Life Sciences, UK) or left untreated as a negative control for 24 hours. DC were harvested from the co-cultures by gentle pipetting using 2 mM EDTA-PBS without affecting adherent CFPAC-1 cells. Harvested DC were either used in other assays or analyzed by flow cytometry to evaluate their activation status.

### Flow cytometry

For mCD40L expression in CFPAC-1 cells transduced with RAdnCD40L, 3 × 10^5^ cells were washed three times with ice-cold PBS buffer and incubated on ice for 20 min with 100 μl of diluted mouse anti-human CD40L-APC conjugate Ab or mouse isotype-APC Ab conjugate (ebioscience, San Diego, CA, USA) or left without treatment as negative control. Cells were then washed three times with ice-cold staining buffer (PBS with 1%BSA and 0.1% NaN3) analysed by flow cytometry. For monocyte-derived DC activation markers, mouse anti-human mAb CD1a- PE-Cy7, CD14-PE, CD40-APC, and HLA-DR-Alexa Fluor 700 were from ebioscience. For mouse anti-human mAb CD86-PE, CD83-APC and CD54-APC, were from BD bioscience, San Jose, CA, USA. Briefly, DC were stained with mouse anti-human mAbs or the isotype control for 20 minutes, cells were washed twice with staining buffer. Flow cytometry was performed by acquiring cells using BD LSR Fortessa cell analyser (BD Bioscience). A minimum of 50,000 HLA-DR + cells were acquired per sample and data were analysed by FlowJo software version X (Tree Star, Ashland, USA)

### IL-10 and IL-12 quantification

CFPAC-1 cells transduced with either 50 MOI RAdMock or RAdnCD40L or un-transduced for 24 hours were co-cultured with iDC at a ratio of 1:2 cells to iDC. The uninfected CFPAC-1 and iDC co-culture were either treated with soluble CD40L (1 µg/ml) (PeproTech) or maturation cocktail or left untreated as a negative control for 48 hours. For IL-10 and IL-12p70 assessment in the culture media, samples were collected at 24 and 48 hours following iDC-CFPAC-1 co-cultures, pre-cleared by centrifugation and assayed by Enzyme-Linked Immune Sorbent Assay (ELISA) kit (eBioscience, Hatfield, UK) according to the manufacturer’s instructions utilizing the automated Modulus Microplate reader (Turner BioSystems, USA).

### Activated DC phagocytic activity

CFPAC-1 cells were labelled with 5 µM carboxyfluorescein diacetate succinimidyl ester (CFSE; Invitrogen, Paisley, UK) for 10 min, washed in 10% FBS culture medium then resuspended at 1 × 10^7^ cells/mL in serum-free RPMI. Necrosis was then induced by six rapid freeze and thaw cycles. CFSE-labelled necrotic cells were incubated with activated DC at a 1:1 ratio for 1 hour at 37 °C. Following two PBS washes, DC were stained with anti HLA-DR AlexaFLuor700-conjugated mAb and uptake of necrotic cell material was analyzed by flow cytometry as a percentage of HLA-DR and CFSE- positive cells in a minimum of 50,000 cells acquired per sample.

### CFSE labeling

Following two washes with PBS, T cells were incubated with 5µmol/L Carboxyfluorescein diacetate succinimidyl ester (CFSE) for 15 minutes at 37 °C before the reaction was terminated by addition of RPMI-1640 containing 10% FBS. Cells were then washed in PBS and resuspended in standard growth medium for subsequent assays.

### *In vitro* priming and proliferation of T cells

For tumour cell lysis, CFPAC-1 cells (10^7^/ml) were lysed by 5 cycles of heating at 42 °C for 10 min then rapid-freezing in liquid nitrogen. The cell lysate preparation was then passed through 0.22 µm syringe driven membrane filter (Millipore). For pulsing pre-activated DC, DC were incubated with CFPAC-1-tumour cell lysate at the ratio of 1:5 (DC: tumour cell equivalent) for 24 hours. CFSE-labelled autologus CD3+ T cells were incubated with DC loaded with tumour cell lysate at a responder to-stimulator (R:S) T-cell/DC ratio of 10:1 at 2 × 10^6^/mL for 5 days or cultured alone as a negative untreated control. Cultures were supplemented with fresh medium containing IL-2 (20 U/ml) and IL-7 (5 ng/ml) (all from PeproTech, London, UK) at day 3. On day 5, we analyzed CD3+ T cells (anti-CD3-pacific blue, BD bioscience, San Jose, CA, USA) for CD8+ T cells (anti-CD8-AlexaFluor 700, BD bioscience, San Jose, CA, USA) with low or negative CFSE staining based on CFSE dilution^20^ as a result of T cell proliferation. A minimum of 100,000 CD3 + cells were analyzed for CD8 + population by flow cytometry. The results were expressed as the percentage of CFSE low or negative cells relative to CD8+ T cells.

### Effector function of DC-activated T cells

*In vitro* expanded T cells obtained from co-culture with DC loaded with tumour lysate for 7 days were assessed for CD107a degranulation and intracellular IFN-γ production. Briefly, 5 × 10^5^ T cells were stimulated for 5 hours in a 96-well plate with irradiated (20 Gy) cell lysate-loaded autologous PBMCs, or left unstimulated as a negative control. GolgiStop (BD Biosciences) and anti-CD107a PE (BD Biosciences) were added 1 hour after stimulation. After 5 hours, cells were stained with anti-CD3-Pacific blue, anti-CD4-FITC and anti-CD8-AlexaFluor 700 for 20 min at 4 °C. Following PBS washing, cells were fixed, permeabilized with Cytofix/Cytoperm solution and stained with anti-IFN-γ APC (BD Biosciences) at 4 °C for 20 min then analysed by flow cytometry. Samples were initially gated for CD3+ CD8+ CD4− T cells then the percentages for CD1017a and IFN-γ positive cells were determined in a minimum of 200,000 CD3+ T cells acquired cells.

### Ethics approval and consent to participate

The use of blood samples for this study was approved by ethical permission from the National Research Ethics Committee (REC: 08/H1011/36). Appropriate approvals and informed written consent for study participation were obtained and the study was performed in accordance with the Declaration of Helsinki.

## Supplementary information

Supplementary Information

Supplementary Information 2

## Data Availability

The datasets generated during the current study are available from the corresponding author on reasonable request.
